# Mucoidy, Quorum Sensing, Mismatch Repair and Antibiotic Resistance in *Pseudomonas aeruginosa* from Cystic Fibrosis Chronic Airways Infections

**DOI:** 10.1371/journal.pone.0012669

**Published:** 2010-09-10

**Authors:** Sofía Feliziani, Adela M. Luján, Alejandro J. Moyano, Claudia Sola, José L. Bocco, Patricia Montanaro, Liliana Fernández Canigia, Carlos E. Argaraña, Andrea M. Smania

**Affiliations:** 1 Centro de Investigaciones en Química Biológica de Córdoba (CIQUIBIC), CONICET, Departamento de Química Biológica, Facultad de Ciencias Químicas, Universidad Nacional de Córdoba, Córdoba, Argentina; 2 Centro de Investigaciones en Bioquímica Clínica e Inmunología (CIBICI), CONICET, Departamento de Bioquímica Clínica, Facultad de Ciencias Químicas, Universidad Nacional de Córdoba, Córdoba, Argentina; 3 Hospital de Niños Santísima Trinidad, Córdoba, Argentina; 4 Hospital Alemán, Buenos Aires, Argentina; Baylor College of Medicine, United States of America

## Abstract

Survival of *Pseudomonas aeruginosa* in cystic fibrosis (CF) chronic infections is based on a genetic adaptation process consisting of mutations in specific genes, which can produce advantageous phenotypic switches and ensure its persistence in the lung. Among these, mutations inactivating the regulators MucA (alginate biosynthesis), LasR (quorum sensing) and MexZ (multidrug-efflux pump MexXY) are the most frequently observed, with those inactivating the DNA mismatch repair system (MRS) being also highly prevalent in *P. aeruginosa* CF isolates, leading to hypermutator phenotypes that could contribute to this adaptive mutagenesis by virtue of an increased mutation rate. Here, we characterized the mutations found in the *mucA*, *lasR*, *mexZ* and MRS genes in *P. aeruginosa* isolates obtained from Argentinean CF patients, and analyzed the potential association of *mucA*, *lasR* and *mexZ* mutagenesis with MRS-deficiency and antibiotic resistance. Thus, 38 isolates from 26 chronically infected CF patients were characterized for their phenotypic traits, PFGE genotypic patterns, mutations in the *mucA*, *lasR*, *mexZ*, *mutS* and *mutL* gene coding sequences and antibiotic resistance profiles. The most frequently mutated gene was *mexZ* (79%), followed by *mucA* (63%) and *lasR* (39%) as well as a high prevalence (42%) of hypermutators being observed due to loss-of-function mutations in *mutL* (60%) followed by *mutS* (40%). Interestingly, mutational spectra were particular to each gene, suggesting that several mechanisms are responsible for mutations during chronic infection. However, no link could be established between hypermutability and mutagenesis in *mucA*, *lasR* and *mexZ*, indicating that MRS-deficiency was not involved in the acquisition of these mutations. Finally, although inactivation of *mucA*, *lasR* and *mexZ* has been previously shown to confer resistance/tolerance to antibiotics, only mutations in MRS genes could be related to an antibiotic resistance increase. These results help to unravel the mutational dynamics that lead to the adaptation of *P. aeruginosa* to the CF lung.

## Introduction

The opportunistic pathogen *Pseudomonas aeruginosa* is the leading cause of morbidity and mortality in CF patients, producing chronic pulmonary infections that can persist over decades [1], [2]. After a period of recurrent infections, CF patients become infected permanently by a single lineage of *P. aeruginosa*, generally acquired from environmental reservoirs [3]. During the establishment of the infection, this single *P. aeruginosa* lineage diversifies into phenotypes specifically adapted to the hostile environment of the CF lung, thus allowing its long-term persistence [4]. These phenotypes possess traits that differ from environmental isolates, but which are commonly observed among CF patients, suggesting a conserved evolutionary pattern in the adaptive process of *P. aeruginosa* to the CF lung [5], [6]. Well-known examples of this diversification process include conversion to the mucoid phenotype [1], [7], loss of the O antigen [8], inactivation of quorum sensing functions [6], [9], [10], loss of motility [11], [12], auxotrophies [13], resistance to antibiotics [14], and a rise in the mutation rate leading to a hypermutator phenotype.

The acquisition of these *P. aeruginosa* CF adaptive phenotypes is mainly based on the occurrence (and further selection) of loss-of-function mutations in specific genes. Among these, the most commonly mutated is *mucA*, which encodes for a negative regulator of the biosynthesis of the exopolysaccharide alginate and whose inactivation is the basis of the conversion to the mucoid phenotype [7]. Another gene, commonly altered even at the early stages of *P. aeruginosa* CF infections, is *lasR*, which is the main quorum sensing regulator controlling the expression of several virulence traits [6], [9], [10]. Moreover, genes which regulate the expression of multidrug-efflux pumps are frequently mutated, such as *mexZ*, a negative regulator of the MexXY-OprM multidrug-efflux pump, whose inactivation increases the resistance to aminoglycoside and other drugs [6], [15].

Another highly prevalent phenotype observed in *P. aeruginosa* isolates from CF patients is the hypermutator phenotype [16]–[19], with hypermutator strains displaying high mutation rates, mainly due to alterations in the genes of the DNA mismatch repair system (MRS) [20]. This phenotype has been reported in CF patients from both Europe and North America [16], [19], [21]–[24], suggesting that this could be a worldwide phenomenon. Moreover, this high prevalence of hypermutators seems to be essentially associated to chronic infections, since it has only very occasionally been observed in either acute infections or environmental samples (≤1%) [19], [24], indicating that it could be linked with the particular genetic adaptive process taking place during the establishment and persistence of *P. aeruginosa* infections. In fact, the link between the acquisition of antimicrobial resistance and hypermutators has been well-established by *in vitro* and *in vivo* approaches [16], [17], [19], [25]–[28]. However, antibiotic resistance is not only a phenomenon restricted to chronic infections, but is also a frequent outcome of acute infections [29]. Working with a *P. aeruginosa* MRS-deficient strain *in vitro*, we previously established an association between hypermutability and both mucoid conversion [30] and *lasR* inactivation [31], [32], both hallmarks of *P. aeruginosa* infections in the CF airways. Similarly, Waine *et al*. [23] observed an association between the hypermutator and the mucoid phenotypes by examining *P. aeruginosa* isolates from adult CF patients with chronic lung infections. Nevertheless, the role of hypermutability in *P. aeruginosa* phenotypic diversification in the natural scenario of a human infection has only very recently been investigated by Mena *et al*. [22], who recently showed that this genetic adaptation is catalyzed by hypermutators. However, the hypermutability was not linked to any particular adaptive trait (including genes involved in the antimicrobial resistance and *lasR*, the latter of which showed a higher number of mutations). Hence, these results show that the involvement of hypermutability in the acquisition of the mutations which drive the adaptive process of *P. aeruginosa* into the CF airways is complex. Also, exactly how *P. aeruginosa* acquires those mutations which enable it to persist into the CF lung environment is still an open question. These issues need to be resolved in order to shed light on the mutational mechanisms involved in *P. aeruginosa* adaptation.

In the present work, we carried out an exhaustive survey and characterization of mutations in *mucA* (mucoid phenotype), *lasR* (quorum sensing deficient phenotype), *mexZ* (multi-drug resistant phenotype) and the MRS genes (hypermutator phenotype) in a collection of *P. aeruginosa* isolates obtained from Argentinean CF patients. *mexZ* turned out to be the most frequently mutated gene followed by *mucA* and *lasR*, but mutations in all these three genes were highly prevalent in our collection. Also, the occurrence of hypermutators among Argentinean CF patients was very high, with hypermutability being a consequence of mutations affecting the MRS genes (mostly the *mutL* gene, but also *mutS*). However, the mutational spectra of each gene were clearly different, suggesting that more than one mechanism could be involved in the mutational process of each gene. Our results show a lack of association between hypermutability and the frequency of adaptive mutations occurring in *mucA*, *lasR* and *mexZ*, and, although hypermutability was associated with an increased resistance to most of the antibiotics tested on *P. aeruginosa* isolates from the Argentinean CF population, this association was not observed for mucoid variants, quorum sensing deficiency or the *mexZ* related multi-drug resistant phenotype.

This is the first detailed characterization of *P. aeruginosa* CF isolates in Latin America which is focused on mucoidy, *lasR-*deficiency, hypermutability and antibiotic resistance, critical issues regarding CF chronic infections, and provides new insights into the mutational mechanisms required for bacterial persistence.

## Results

### Phenotypic and genotypic characterization of the *P. aeruginosa* isolates

Thirty-eight *P. aeruginosa* isolates were collected from the sputum of twenty-six CF patients during the course of chronic pulmonary infection (see Material and Methods). The samples were obtained from child/teenage patients (18 isolates, 12 patients) as well as from adult patients (20 isolates, 14 patients) obtained at two Hospitals located in different Argentinean cities. In order to avoid duplications, two isolates from the same patient were considered different if they fulfilled at least one of the following conditions: 1) they were isolated with a separation period of at least one year; 2) they were obtained on the same date, but showed different colony morphotypes (see Material and Methods).

We characterized all *P. aeruginosa* isolates based on their mucoidy, iridescence at white light exposure, pigmentation, and colony size. As shown in Table 1, this first phenotypic characterization displayed the typical colony variant diversification described for pulmonary chronic infection isolates, which differed from the monomorphic variants isolated from *P. aeruginosa* acute infections [5], [6].

**Table 1:** Genotypic and morphotypic characterization of the 38 *P. aeruginosa* CF isolates.

Patient	Isolate	Genotype^a^	Phenotype					Source	Year of isolation
			Mucoidity	SCV	Pigmentation	Iridescence	Mutation Frequency^b^		
1	a	ND	+	-	-	-	**(1.3**±**0.7)×10^−5^**	HNC	2004
	b	A1	+	-	-	-	**(1.6**±**0.7)×10^−5^**	HNC	2007
	c	A1	+	+	-	-	(2.7±1.9)×10^−9^	HNC	2007
2	a	A1	-	+	-	+	**(1.0**±**0.4)×10^−5^**	HNC	2007
	b	A2	+	-	-	-	(1.7±1.4)×10^−9^	HNC	2007
3	a	B1	+	-	-	-	**(1.0**±**0.7)×10^−5^**	HNC	2004
	b	B1	-	+	-	-	**(1.6**±**0.3)×10^−6^**	HNC	2007
	c	B1	+	-	+ (green)	-	**(2.8**±**1.6)×10^−7^**	HNC	2008
4	a	C1	+	-	-	-	(2.8±1.4)×10^−8^	HNC	2007
	b	C1	-	-	-	-	(9.0±6.3)×10^−9^	HNC	2007
5	a	D1	+	-	-	-	**(4.5**±**2.6)×10^−6^**	HNC	2007
6	a	E1	-	-	-	-	(6.4±4.5)×10^−9^	HNC	2007
7	a	Q1	-	-	+ (green)	-	(2.9±0.8)×10^−8^	HNC	2007
8	a	R1	+	-	-	-	(2.2±0.5)×10^−8^	HNC	2007
9	a	F1	+	-	-	-	**(3.5**±**0.3)×10^−6^**	HNC	2004
10	a	L1	+	+	-	-	**(6.5**±**3.8)×10^−6^**	HNC	2008
11	a	N1	+	-	-	-	(1.0±0.5)×10^−9^	HNC	2008
12	a	O1	+	-	-	-	(3.9±2.1)×10^−9^	HCN	2008
13	a	G1	-	-	-	+	(4.1±0.3)×10^−9^	HABA	2006
14	a	H1	-	-	+ (green)	-	(5.0±3.2)×10^−9^	HABA	2006
15	a	S1	-	+	+ (red)	-	(1.1±0.6)×10^−7^ c	HABA	2006
16	a	I1	+	-	+ (green)	-	(6.7±1.8)×10^−9^	HABA	2006
17	a	J1	-	-	+ (green)	-	(2.6±1.5)×10^−9^	HABA	2006
	b	J1	+	-	-	-	**(6.0**±**2.8)×10^−6^**	HABA	2006
18	a	K1	+	+	-	-	(5.5±2.1)×10^−9^	HABA	2006
	b	K1	-	+	-	-	(2.9±2.3)×10^−8^	HABA	2006
	c	K1	+	-	-	-	(1.2±1.1)×10^−9^	HABA	2008
	d	K1	-	+	-	+	**(1.3**±**1.2)×10^−6^**	HABA	2008
19	a	M1	+	-	-	-	**(2.6**±**0.4)×10^−6^**	HABA	2006
20	a	P1	+	-	+ (green)	-	(9.0±4.0)×10^−10^	HABA	2008
	b	P1	-	-	+ (green)	-	**(2.2**±**1.6)×10^−6^**	HABA	2008
21	a	T1	+	+	-	-	**(1.5**±**0.3)×10^−6^**	HABA	2008
	b	T1	-	+	-	+	**(1.1**±**0.9)×10^−6^**	HABA	2008
22	a	U1	+	-	-	-	**(3.7**±**2.2)×10^−6^**	HABA	2008
23	a	V1	-	-	+ (green)	-	(1.9±1.3)×10^−9^	HABA	2008
24	a	W1	-	-	-	+	(4.0±2.0)×10^−10^	HABA	2008
25	a	X1	-	-	-	+	(1.1±0.2)×10^−7^ c	HABA	2008
26	a	Y1	+	-	-	-	(2.3±1.7)×10^−9^	HABA	2008

a All isolates were genotyped by PFGE using the *SpeI* enzyme.

b Mutation frequency was measured as the occurrence of spontaneous resistance to rifampin 100 µg/ml.

c Isolates 15a and 25a with mutation frequencies nearly under the breakpoint (≥2×10^−7^) were discarded to be *mutS* or *mutL* deficient strains by gene sequencing (see Table 2).

SCV, Small Colony Variant; ND, not determined; HNC, Hospital de Niños de Córdoba; HABA, Hospital Alemán de Buenos Aires.

Those isolates with mutation frequencies ≥2×10^−7^ were defined as hypermutators and indicated by bold type.

Mutation frequencies for PAO1 reference strain and MPAOMS and MPAOML hypermutator strains were (2.4±1.3)×10^−8^, (2.8±1.6)×10^−6^ and (1.3±0.6)×10^−6^ respectively.

In order to explore the extent of clonality and the epidemiology of the *P. aeruginosa* collection, we carried out a PFGE analysis of the 38 CF isolates. Each patient carried a single unique genotype that persisted over time, even for those isolates that were obtained from the same patient over several years (patients 3 and 18). Moreover, the strains from different patients were epidemiologically unrelated (Table 1), indicating that the infection in each patient was established independently by bacterial lineages from different environmental reservoirs.

Exceptions to this distribution were the isolates from patients 1 and 2, which demonstrated a closely related PFGE pattern (Table 1). This exceptional case may indicate a very low prevalence (5%) of transmission among patients in the Argentinean population.

### *P. aeruginosa* hypermutator strains in the Argentinean CF population

In order to establish the prevalence of hypermutator strains in the Argentinean *P. aeruginosa* CF isolates, we determined the frequency of mutation to rifampin resistance for the 38 isolates. Although this assay only allows the detection of base substitutions in the *rpoB* gene [33], it is widely used as an efficient method to detect MRS deficient strains [19], [34].

Thus, the results were compared with those obtained for the PAO1 reference strain (2.4×10^−8^±1.3×10^−8^) as well as with those from the MPAOMS (2.8×10^−6^±1.6×10^−6^) and MPAOML (1.3×10^−6^±0.6×10^−6^) hypermutator mutant strains. In this sense, clinical isolates were considered hypermutators when their mutation frequencies were ≥2×10^−7^, with this being an arbitrary value which has been previously established to be reached/surpassed mostly by MRS-defficient strains [19]. As observed in Table 1, 16 hypermutators were identified among the 38 *P. aeruginosa* isolates (42%), with these hypermutable *P. aeruginosa* strains being obtained from 12 patients (46%). It is important to mention that the prevalence of *P. aeruginosa* hypermutator strains was very high in the adult population as well as in the child/teenaged population.

### Molecular characterization of the hypermutator strains

It has been described that the stable hypermutators found among *P. aeruginosa* CF isolates are commonly deficient in the genes belonging to the MRS, particularly *mutS* [18], [20]. Thus, we explored the genetic bases for the hypermutability of the 16 *P. aeruginosa* hypermutator isolates by carrying out gene complementation assays as well as sequencing of the *mutS* and *mutL* genes. As shown in Table 2, 15 hypermutator isolates (94%) were confirmed to be defective in the MRS, of which 6 isolates showed a loss of function in the *mutS* gene, and 9 in the *mutL*. This observation differs from previous studies in which *mutS* was shown to be the main target for the acquisition of a stable hypermutable state [18], [20]. For the remaining hypermutator isolate (3c), although genetic complementation was not achieved due to this isolate showing a natural resistance to gentamicin (not shown), a region of its *mutS* gene could not be amplified by PCR, suggesting that this strain could also be another *mutS*-deficient isolate. Mainly frameshift mutations (62.5%, see below) were responsible for the hypermutator phenotype in both the *mutS* and *mutL* genes, including deletions and insertions (Table 2). Furthermore, in three hypermutator isolates, missense mutations accounted for the inactivation of the MRS genes: isolate 5a had a single point mutation leading to a change in amino acid 493 from tyrosine to proline (T493P), located in the clamp domain IV of the MutS protein; isolate 10a contained a non-synonymous mutation in *mutS*, G618S, one of the ADP-contacting residues of ATPase domain V [35] that is highly conserved in all ABC ATPases inhibited by vanadate [36]; and the remaining isolate 2a had a single point mutation in *mutL*, which produced a change in amino acid 625 from leucine to glutamine (L625Q). This amino acid is one of the last 10 C-terminal residues that abolishe MutL homodimerization upon deletion, a phenomenon that has a key function in communicating mismatch recognition by MutS to downstream repair processes. To our knowledge, these three missense mutations have not been previously reported.

**Table 2:** Mutations in the *mucA*, *lasR* and *mexZ* genes of the 38 *P. aeruginosa* CF isolates.

Isolate	*mucA*	*lasR*	*mexZ*	MRS genes
1a	C→T at 349 (TAA at 349)	A→G at 587 (E196G)	C→G at 151 (H51D)	*mutS*, 11 bp del at 1555 (TAA at 1854); A→G at 1276 (T426A)
1b	C→T at 349 (TAA at 349)	A→G at 587 (E196G)	C→G at 151 (H51D)	*mutS*, 11 bp del at 1555 (TAA at 1854); A→G at 1276 (T426A)
1c	1 bp del at 426 (TGA at 440)	NF	1 bp del at 261 (TAA at 332)	ND
2a	NF	1 bp del at 308 (TGA at 341)	1 bp del at 242 (TAA at 332)	*mutL*, T→A at 1874 (L625Q)
2b	1 bp del at 426 (TGA at 440)	NF	1 bp del at 261 (TAA at 332)	ND
3a	1 bp del at 426 (TGA at 440)	NF	NF	*mutL*, 2 bp ins at 619 (TGA at 784)
3b	1 bp del at 426 (TGA at 440)	NF	NF	*mutL*, 11 bp del at 619 (TGA at 782)
3c	NF	NF	NF	*mutS*, NA
4a	1 bp del at 426 (TGA at 440)	NF	10 bp del at 595	ND
4b	1 bp del at 426 (TGA at 440)	NF	10 bp del at 595	ND
5a	1 bp del at 426 (TGA at 440)	NF	NF	*mutS*, A→C at 1477 (T493P)
6a	NF	NF	2 bp del at 60	ND
7a	1 bp del at 426 (TGA at 440)	A→G at 626 (N209S)	T→C at 110 (L37P)	ND
8a	C→T at 352 (TAG at 352)	NF	10 bp del at 184	ND
9a	C→T at 349 (TAA at 349)	2 bp del at 602 (TAG at 617)	14 bp del at 340	*mutL*, 17 bp del at 1240 andG→T at 1632 (TGA at 1632)
10a	C→T at 340 (TAG at 340)	NF	11 bp del at 328	*mutS*, G→A at 1852 (G618S)
11a	1 bp del at 426 (TGA at 440)	NF	1 bp deletion at 609	ND
12a	NF	NF	NF	ND
13a	NF	NA	A→G at 633 (TAG 211 W)	ND
14a	NF	NF	A→G at 34 (T12A); G→A at 44 (G15D)	ND
15a	NF	NF	NA	NF
16a	1 bp del at 201 (TGA at 284)	NF	11 bp del at 365	ND
17a	NF	NF	38 bp del at 417	ND
17b	NF	NF	13 bp del at 340	*mutS*, 2 bp ins at 1611 (TGA at 1687); T→C at 986 (L329P)
18a	1 bp del at 339 (TGA at 386)	NF	4 bp del at 595	ND
18b	1 bp del at 339 (TGA at 386)	NF	4 bp del at 595	ND
18c	1 bp del at 430 (TGA at 440)	NF	15 bp del at 60 (ΔRVFLE at 22)	ND
18d	G→T at 421 (TAG at 421)	C→T at 617 (A206V)	NF	*mutS*, 1 bp del at 1171 (TGA at 1250)
19a	1 bp del at 426 (TGA at 440)	NF	368 bp del at 248	*mutL*, 5 bp ins at 627 (TGA at 782)
20a	C→T at 367 (TAG at 367)	T→G at 645 (I215M)	C→T at 187 (C63R)	ND
20b	NF	T→G at 645 (I215M)	C→T at 187 (C63R); C→T at 283 (TAG at 283)	*mutL*, 96 bp dup at 75 (TGA at 378)
21a	NF	C→T at 221 (P74L)	21 bp del at 631	*mutL*, 4 bp del at 1475
21b	NF	C→T at 221 (P74L)	21 bp del at 631	*mutL*, 4 bp del at 1475
22a	1 bp del at 358 (TGA at 386)	C→T at 280 (TAG at 280)	12 bp del at 425 (ΔRAVE at 143; R142Q)	*mutL*, C→T at 1474 (TGA at 1474)
23a	G→C at 304 (G102R)	A→G at 575 (K192R)	T→G at 413 and C→A at 414 (L150R); 7 bp del at 451	ND
24a	NF	C→A at 317 (TGA at 315)	C→A at 494 (TAG at 493)	ND
25a	NF	A→T at 697 (N233Y)	NF	NF
26a	1 bp del at 201 (TGA at 284)	NF	12 bp del at 383 (ΔPLEK at 128)	ND

NF, no mutation found; ND, not determined; NA, not PCR amplified; del, deletion; ins, insertion; dup, duplication.

Finally, it is interesting to highlight the results obtained for patients 1 and 3. From patient 1, we isolated a previous hypermutator strain in 2004 (1a) and a subsequent hypermutator strain in 2007 (1b) (Table 1). Both these isolates contained the same 11 bp deletion in MutS and an identical single point mutation (T426A), suggesting that the same hypermutator lineage was maintained over three years of chronic infection. On the other hand, the hypermutator isolates from patient 3 obtained in 2004 (3a) and 2007 (3b) harbored two different frameshifts, produced by 11 bp and 2 bp deletions inactivating *mutL*, respectively. In addition, a third hypermutator isolate (3c) was obtained from the same patient in 2008, which possessed an unaltered *mutL* sequence but which failed to amplify a region of the *mutS* gene. This result indicates that during four years of chronic infection in patient 3, three different hypermutator lineages emerged, suggesting a strong selection for hypermutability in the CF lung.

### Screening of mutations in the *mucA*, *lasR* and *mexZ* genes

The coding sequences of the *mucA*, *lasR* and *mexZ* genes were analyzed in our collection of isolates to score for mutations that could be involved in the phenotypic switches to mucoidy, deficiency in the quorum sensing and antimicrobial (aminoglycoside) resistance respectively, three phenotypes considered to be characteristics of *P.aeruginosa* chronic CF infections. Thus, only nonsynonymous mutations which are expected to cause partial or complete loss of function in these genes were considered in the analysis (see Material and Methods).

As shown in Table 2, *mexZ* was the most frequently mutated gene in our collection of isolates, with 30 out of 38 isolates harboring mutations in this gene (79%). This was followed by *mucA*, which showed mutations in 24 of the 38 isolates (63%), and finally *lasR*, which was mutated in 15 isolates (39%). Taken together, these results and the data from the MRS gene mutational inactivation (42%) confirm that the occurrence of mutations in all the analyzed genes was highly prevalent among Argentinean CF patients.

### Mutational spectra are remarkably different among *mucA*, *lasR*, *mexZ* and MRS genes

**Figure 1:**
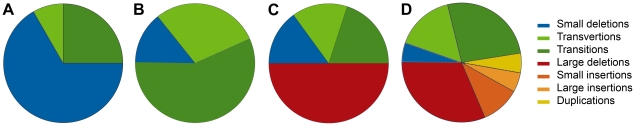
Mutational spectra of *mucA*, *lasR*, *mexZ* and MRS genes observed in *P. aeruginosa* isolates obtained from Argentinean CF patients. Pie charts indicate the observed percentage for each kind of mutation respect to the total number of mutations occurring in (A) *mucA*, (B) *lasR*, (C) *mexZ* and (D) MRS genes. The analyses on MRS genes include mutations observed in *mutS* and *mutL.*

Next we examined the mutational spectrum produced in each gene. Interestingly, these mutational spectra presented large differences among *mucA*, *lasR*, *mexZ*, and *mutS/mutL*, which were particular to each gene (Figure 1). In *mucA*, small deletions prevailed (64.7%) with the rest of the mutations consisting of different kinds of transitions (25%) and transversions (8.3%) (Figure 1A). Notably, both frameshift mutations and substitutions generated premature stop codons in 92% of the cases (Table 2), indicating the particular way in which *mucA* was mutated. In *lasR*, missense substitutions prevailed (86%), with a 2∶1 ratio for transitions (57%) over transversions (29%) (Figure 1B), which mainly produced changes in the amino acid sequences (Table 2). In contrast with *mucA*, only 14% of mutations in *lasR* were small deletions (Figure 1B). *mexZ*, on the other hand, showed mostly deletions (65%), of which there was a high prevalence of large deletions (>4 bp) (50%) respect to small deletions (1–4 bp) (15%), followed by transitions (20%) and transversions (15%) (Figure 1C). However, in *mexZ*, almost 15% of mutations generated premature stop codons (Table 2). As mentioned above, mostly frameshift mutations (62.5%) were responsible for *mutS* and *mutL* inactivation (Table 2), including mainly large (31.5%) but also small deletions (5.3%), small insertions (10.5%) large insertions (5.3%) and duplications (5.3%), thus showing a wider mutational spectrum (Figure 1D).

By comparing the mutational spectra obtained in the Argentinean isolates with those reported in other studies [6], [22], [37]–[39], we observed that our results coincided with those previously described for *mucA*, *lasR*, *mexZ* and MRS, indicating that each gene has a particular way of being mutated. Taken together, these observations suggest that different mechanisms of mutagenesis are responsible for the loss-of-function mutations in the analyzed genes.

### Mutations in the *mucA*, *lasR* and *mexZ* genes are not linked to hypermutability in the Argentinean CF population

Theoretical studies suggest that, under stressful conditions, selective pressure favors hypermutator strains over nonmutator strains [40]–[42]. In the particular case of *P. aeruginosa* chronic lung infection, it has been proposed that the proliferation of the hypermutators in the population infecting an individual patient may be sustained by their association (hitchhike) with mutations adaptive to growth in the lung [19], [20]. Related to this, in two previous recent works, we showed *in vitro* that the MRS loss-of-function was a key determinant in targeting *mucA* for mucoid conversion and *lasR* for quorum sensing deficiency [30]–[32].

**Figure 2:**
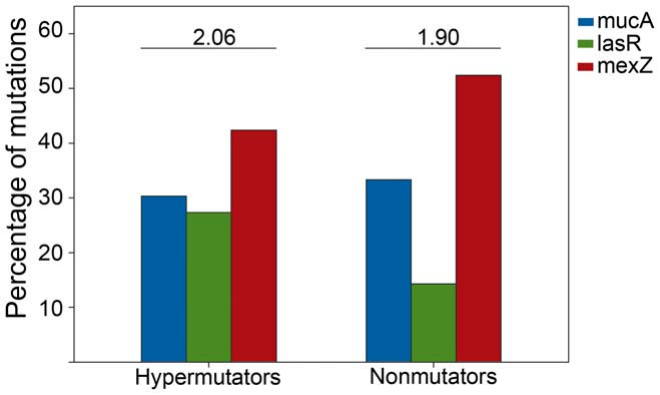
Distribution of the number of mutations among *mucA*, *lasR* and *mexZ* genes in hypermutator and nonmutator isolates. The number of mutations occurring in *mucA* (blue bars), *lasR* (green bars) and *mexZ* (red bars) is expressed as a percentage respect to the total number of mutations found in hypermutator and nonmutator isolates. Above the bars, the total number of mutations per isolate for the hypermutator and nonmutator subpopulations is indicated.

In order to investigate if an association could be established between hypermutability and the mutations observed in the *mucA*, *lasR* and *mexZ* genes in the Argentinean CF isolate collection, we analyzed the proportion of mutations that occurred in each gene in the hypermutator and nonmutator isolates. As shown in Figure 2, we observed that in the hypermutator isolates, the proportion of mutations that occurred in the *mexZ* and *mucA* genes was not significantly different to that observed in the nonmutator isolates (*P* = 0.17 and *P* = 1.00 respectively). In the case of *lasR*, we saw that among the hypermutators, the number of mutations per isolate was higher (0.56) than nonmutators (0.27), although this difference was not statistically significant (*P* = 0.09), indicating that, as in the case of *mexZ* and *mucA*, it was not possible to establish an association between the occurrence of mutations in *lasR* and the hypermutator phenotype. Furthermore, as shown in Figure 2, the number of mutations per hypermutator isolate (2.06) was not higher than that observed per nonmutator isolate (1.90).

We now investigated if hypermutability could be associated with any particular kind of mutations in *mucA*, *lasR* and *mexZ* genes. It was observed that although the hypermutator isolates showed a tendency towards substitutions (53%), which were either transversions or transitions, with nonmutator isolates displaying a higher number of deletions (62%) (mainly small deletions between 1 to 4 bp), there was not a statistically significant difference between hypermutators and nonmutators with respect to the kind of mutations occurring in the analyzed genes. Furthermore, whatever the type of mutations, their distribution among any particular analyzed gene in hypermutators and nonmutators isolates did not show any statistically significant differences (Figure 2). Therefore, it was not possible to establish any association between hypermutability and the nature of the mutations that occurred in *mucA*, *lasR* and *mexZ* during the process of chronic lung infection, suggesting that in the population analyzed in this work, hypermutation was not linked (hitchhiked) to any of the specific adaptive traits studied.

### Hypermutability is associated to antimicrobial resistance mainly through the presence of resistant mutant subpopulations (RMS)

As mentioned above, several reports have established a link between antimicrobial resistance and the occurrence of hypermutators. In fact, it has been observed that hypermutator *P. aeruginosa* strains isolated from CF patients not only possess a substantially higher antibiotic resistance than nonmutator isolates [19], but also increase the rate of acquisition of new antibiotic resistance [25]. Thus, in order to investigate the association between hypermutability and antibiotic resistance in the Argentinean *P. aeruginosa* CF isolates, we tested the 38 isolates by measuring the inhibition zone diameters and scoring for RMS within the inhibition zones of five different antimicrobial agents (ceftazidime, ciprofloxacin, imipenem, meropenem and tobramycin). As shown in Table 3, the proportion of hypermutator strains of *P. aeruginosa* with detectable levels of ciprofloxacin and ceftazidime resistance was significantly higher than the proportion observed in non-mutator strains (*P* = 0.02 and *P* = 0.02 respectively), indicating that in the Argentinean CF population, hypermutability correlated with ciprofloxacin and ceftazidime resistance. In fact, 62.5% of the hypermutators were ciprofloxacin resistant compared to a proportion of 22.7% found among nonmutators. Although a smaller proportion of hypermutators (25%) were ceftazidime resistant, all nonmutator strains were susceptible to this antibiotic (Table 3). The prevalence of antibiotic resistant strains for hypermutator and nonmutator isolates for imipenem, meropenem, and tobramycin was biased towards the hypermutators, although this was not statistically significant (*P* = 0.06, *P* = 1.00 and *P* = 0.61 respectively). Nevertheless, it is important to point out that only one isolate (isolate 19a) that was a hypermutator showed resistance to all the antibiotics tested (Table 3).

**Table 3:** Antibiotic disk diffusion test and quantification of the resistant mutant subpopulations of the 38 *P. aeruginosa* CF isolates**.**

Isolate	MRS genes^a^	Ceftazidime		Ciprofloxacin		Imipenem		Meropenem		Tobramycin	
		DD	RMS	DD	RMS	DD	RMS	DD	RMS	DD	RMS
1a	-	S	+++	S	++	S	+++	S	+++	S	++
1b	-	S	+++	I	++	S	+++	S	+++	R	*
1c	+	S	++	S	-	S	-	S	++	S	-
2a	-	R	*	R	*	S	+++	S	+++	S	+++
2b	+	S	+	S	-	S	-	S	+	S	-
3a	-	I	++	I	-	S	+++	S	+++	I	+++
3b	-	R	*	I	++	I	+++	S	+++	I	+++
3c	-	S	+++	S	+++	S	+++	S	+++	S	++
4a	+	S	-	S	-	S	-	S	-	S	-
4b	+	S	-	S	-	S	-	S	-	S	-
5a	-	S	-	R	*	S	++	S	+++	S	+
6a	+	S	-	S	-	S	-	S	-	S	-
7a	+	S	-	I	-	S	-	S	-	S	-
8a	+	S	-	R	*	S	+++	S	+++	S	-
9a	-	S	++	S	+++	S	+++	S	+++	S	+++
10a	-	S	++	S	++	S	+++	S	-	S	+++
11a	+	S	-	S	-	S	-	S	-	R	*
12a	+	S	-	S	-	S	-	S	-	S	-
13a	+	S	-	R	*	S	-	S	-	R	*
14a	+	S	-	S	-	S	-	S	-	S	-
15a	+	S	-	S	-	S	-	S	-	S	-
16a	+	S	-	S	-	S	-	S	-	S	-
17a	+	S	-	I	-	R	*	I	-	S	-
17b	-	S	-	S	-	I	+++	S	++	S	+
18a	+	S	-	S	-	S	-	S	-	S	-
18b	+	S	-	R	*	S	-	S	-	S	-
18c	+	S	-	S	-	S	-	S	-	S	-
18d	-	S	+++	R	*	R	*	S	+++	R	++
19a	-	R	*	R	*	R	*	R	*	R	*
20a	+	S	-	S	-	S	-	S	-	S	-
20b	-	S	+++	R	*	R	*	S	+++	S	+++
21a	-	S	+++	R	*	S	++	S	+++	S	+++
21b	-	S	+++	R	*	S	+++	S	+++	S	+++
22a	-	S	-	S	-	S	++	S	+++	S	+++
23a	+	S	-	S	-	S	-	S	++	S	-
24a	+	S	-	S	-	S	-	S	-	S	-
25a	+	S	-	S	-	S	-	S	-	S	-
26a	+	S	-	S	-	S	-	S	-	S	-

a MRS genes was considered positive (+) when the sequences of *mutS* and *mutL* genes were wild type and negative (−) when were mutated.

DD, diffusion diameter zone; RMS, resistant mutant subpopulation: +<10 mutants; ++ 10 to 100 mutants; +++ >100 mutants.

Zone diameter interpretative criteria was according to CLSI: S, susceptible; I, intermediate resistance; R, resistant.

*lack of diffusion zone that prevents the resistant mutant subpopulation to be quantified.

By quantifying the emergence of the resistant mutant subpopulation (RMS) [43], we observed that most of the hypermutator isolates showed RMS within the inhibition zones of the five antibiotics tested. In contrast, RMS was rarely detected when the nonmutator strains were tested for the same antibiotics. In fact, every hypermutator isolate of this study showed RMS for at least three different antibiotics, with the proportion of hypermutator *P. aeruginosa* strains that showed RMS being significantly higher than the non-mutator strains (*P<*0.001) (Table 3). These observations are in agreement with a previous study, in which the disk diffusion test was proposed as a reliable method for detecting hypermutator strains from clinical isolates [43].

Finally, it is important to remark that in the Argentinean population, the disk diffusion tests showed similar results for the child/teenage and the adult groups of CF patients.

### Antimicrobial resistance is not linked to mucoidy, *lasR* inactivation or *mexZ* related multi-drug resistant phenotype

**Figure 3:**
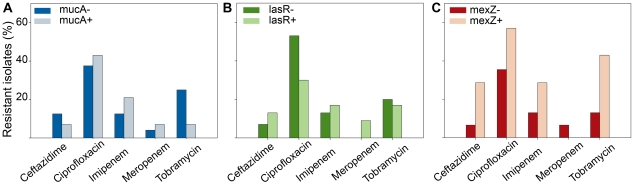
Association between antibiotic resistance and inactivation of *mucA*, *lasR* and *mexZ.* (A) Differences in antibiotic resistance in the *P. aeruginosa* isolates harboring mutations in *mucA* (mucA-) respect to those without mutations in *mucA* (mucA+). Data is expressed as the percentage of resistant isolates to ceftazidime, ciprofloxacin, imipenem, meropenem and tobramycin in mucA- and mucA+ isolates. (B) The same analysis was carried out in lasR- and lasR+ isolates and (C) mexZ- and mexZ+ isolates. In the three genes, the observed differences were not significant (Fisher's exact test) for any of the tested antibiotics.

Previous studies have reported an enhanced resistance to antibiotics for mucoid strains of *P. aeruginosa* isolated from sputa of CF patients [44]. Also, it has been proposed that *mexZ* inactivation is involved in the development of stable aminoglycoside resistance among CF strains of *P. aeruginosa* [15]. An increased tolerance to β-lactam has been attributed to inactivation of *lasR*, which was not sufficient to alter the MIC, but resulted in a greater production of spontaneous resistant colonies [9]. Moreover, it has been recently observed that *lasR* loss-of-function mutations confered resistance to ciprofloxacin and tobramycin when oxygen was limited and there was increased nitrate utilization [45]. Related to this, we investigated if the inactivation of *mucA*, *lasR* or *mexZ* could be associated with an altered antibiotic resistance in our collection. As shown in Figure 3, antibiotic resistance to β-lactams ceftazidime, imipenem and meropenem, as well as flouroquinolone ciprofloxacin and aminoglycoside tobramycin was not associated with the mutagenic inactivation of *mucA*, *lasR* or *mexZ*. Moreover, scoring for RMS within the inhibition zones of the five antimicrobial agents tested showed that the presence of resistant mutants was not a phenomenon related to the inactivation of any of these three regulators, not even when the *lasR* mutants were tested for β-lactam antibiotics (not shown).

## Discussion

*P. aeruginosa* establishes chronically in the CF airways by a diversification process that allows fine tuning-adaptation and long-term survival within the host. Since this process is mainly mutagenic [6], a better understanding of the means by which *P. aeruginosa* acquires these mutations may help to identify the functions required for bacterial persistence.

In this study, we carried out a survey and a molecular characterization of the mutations that occurred in the genes which had been previously described as important determinants for the adaptation of *P. aeruginosa* to the CF lung, due to their being the most mutated among CF patients [6]. The *mucA*, *lasR* and *mexZ* genes as well those implicated in the inactivation of MRS, *mutS* and *mutL*, were analyzed in a collection of *P. aeruginosa* isolates obtained from Argentinean CF patients, with this constituting the first study of this nature in Latin America. According to our results, mutations in the first three genes were highly prevalent in the Argentinean population, with *mexZ* being the most frequently mutated followed by *mucA* and *lasR* (Table 2). In addition, a high prevalence of hypermutator strains was observed in our collection (Table 1), in agreement with what has been previously reported in other geographical regions [16]–[19]. All the hypermutator strains were the result of a deficiency in MRS, and despite most reports have described mutations in the *mutS* gene as the main cause of hypermutability, followed by mutations in *mutL* and *uvrD* [18], [20], in our collection, mutations in *mutL* were actually the most frequent cause of MRS deficiency, with the *mutS* gene alterations playing a secondary role (Table 2). It is worth mentioning that, based on the defined mutation frequency breakpoint (≥2×10^−7^), our analysis was focused on strong MRS deficient hypermutators, not considering weak mutators (usually with alterations in the *mutT*, *mutY* and *mutM* genes of the GO system) which can also be found among the CF *P. aeruginosa* isolates [16], [19], [46].

It is proposed that the high prevalence of stable hypermutator strains of *P. aeruginosa* in the airways of CF patients is a consequence of their second-order selection with other adaptive mutations, since a higher mutation rate would increase their probability to be acquired [42]. Related to this, an association between hypermutability and mutations that confer antibiotic resistance has been established by several reports [21], [25], [26], [43], [47]. Here, we also observed that MRS-deficiency was linked to resistance to several antibiotics (Table 3), thus confirming that in our collection, hypermutability was a key factor in the generation of mutation-mediated resistance. However, antibiotic resistance could not be associated to the mucoid phenotype [44] and the *lasR* quorum sensing-deficient phenotype [9], [15] (Figure 3). Even inactivation of MexZ was not linked to antibiotic resistance in our collection of isolates. This last observation results intriguing, since inactivation of this gene leads to the overproduction of the MexXY efflux system which is one of the most frequently reported mechanisms of acquiring resistance to aminoglycoside and other drugs among CF isolates [26] (Figure 3). Our results suggest that antibiotic resistance due to overproduction of MexXY may require the existence of additional regulatory mechanisms other than the inactivation of *mexZ* [15], [48], [49]. Thus, it is possible to suggest that those mutations linked to hypermutability, but not the inactivation of *mucA/lasR/mexZ*, may confer a level of antibiotic resistance detectable by disk diffusion, which is the methodology used in this study.

In a previous recent work, we determined *in vitro* that MRS-deficiency accelerates the acquisition of *mucA* [30] and *lasR* [31], [32] mutations, which led us to the hypothesis that such an association may also be occurring *in vivo*. In line with this, Waine *et al* [23] described a link between the hypermutator and mucoid phenotypes in *P. aeruginosa* isolates obtained from CF patients. However, in the present study, by scoring for mutations in hypermutator and nonmutator CF isolates of our collection, no association could be established between hypermutability and mutagenesis, not only for *mucA* but also for the *lasR* and *mexZ* mutations. A possible explanation for this discrepancy is that in Waine *et al*., the phenomenon was not characterized at the genetic level, and that the reversion phenomenon of the mucoid to a nonmucoid phenotype, which is highly frequent in isolates recovered from CF patients [38], was not evaluated. In fact, this analysis on our collection showed that 37.5% of the non-mucoid isolates harbored mutations in *mucA*, indicating that they had previously been mucoid before reverting to the phenotype. Furthermore, contrary to our isolates which were clonally different, almost half of the isolates in Waine *et al*. corresponded to epidemic clones, a feature that increases dissimilarities among collections.

It has been recently shown that hypermutator strains accelerate the mutagenic genetic adaptation thus enhancing the accumulation of new mutations [22]. However, no differences were observed between hypermutators and nonmutators isolates with respect to the distribution of mutations among several sequenced genes, suggesting that the hypermutability had a widespread effect on genes during the adaptation process in the lung, but was not linked to any specific adaptive trait, even to antibiotic resistance. [22]. Consistent with this antecedent, we observed similar mutational spectra in the *mucA*, *lasR* and *mexZ* genes of hypermutator and nonmutator isolates. However and most interestingly, these mutational spectra vary in a gene dependant manner, with *mucA* being dominated by small deletions (1 bp), *lasR* by base substitutions and *mexZ* by large deletions (>4 bp). This observation is intriguing, since it suggests the involvement of different mechanisms of mutagenesis, possibly operating simultaneously, during the process of CF chronic infection. *mucA* and *lasR*, but not *mexZ* mutational spectra coincide well not only with the spectrum generated by a MRS deficiency [39], [50] but also with the activity of SOS-inducible error prone polymerases (i.e., Pol IV) [51]. Therefore, it can be postulated that the induced hypermutability due to the stressful milieu of the CF lung plays an important role in mutagenesis, thereby contributing to the genetic adaptation of *P. aeruginosa* to this environment. Consistent with this hypothesis, we have recently shown that Pol IV activity is an essential ingredient to establish *mucA* as the main target for mutagenesis in mucoid conversion, with this factor having a prominent role in the generation of -1 deletion in a monomeric simple sequence repeats of five Gs (G^5^-SSR_426_) one of the most prevalent mutations among CF mucoid isolates [30]. In fact, elimination of G^5^SSR_426_ by site directed mutagenesis not only significantly reduces the mutation frequency in *mucA*, but also makes *mucA* to be no longer the major pathway for mucoid conversion [52]. In a very recent work, carried out at the same time as our study, a lack of association between stable hypermutability and *mucA* and *lasR* mutations was also found [53]. According to this work, mucoid isolates as well as *lasR*-deficient isolates occur prior to the appearance of hypermutator strains, which had previously been shown to accumulate at later stages of the infection [6], [47]. Thus, it is possible that mutations in these genes might be more important for survival at the earlier stages of chronic infection, when the effect of hypermutators is still poor. Taken all together, our results clearly suggest that mutations which inactivate *mucA*, *lasR* and *mexZ* arise by different mechanisms in each gene, with these being independent of MRS-deficiency. However, further research is required to achieve a better understanding of the complex and variable mutagenic strategies used by *P. aeruginosa* to continuously adapt to the environment of the CF lung.

## Materials and Methods

### *P. aeruginosa* isolates from CF patients

Thirty-eight *P. aeruginosa* isolates, representing different colony morphotypes (with respect to mucoidy, iridescence, colony size and pigmentation), were collected from sputum samples of 26 Argentinean CF patients with chronic *P. aeruginosa* lung infection (with at least four years of continuous isolation of *P. aeruginosa*). These patients were either children/teenagers or adults, who had been treated at two local Hospitals: Hospital de Niños Santísima Trinidad (Córdoba city) (12 patients; age range, 5 to 20 years; mean age, 13±3.5) or Hospital Alemán (Buenos Aires city) (14 patients; age range, 15 to 43 years; mean age, 27±7). The *P. aeruginosa* reference strain PAO1 and hypermutable strains MPAOMS and MPAOML were used as controls (Table 4). All strains were maintained and stored at −70°C in glycerol stocks. For the different assays, strains were routinely subcultured on Luria Bertani (LB) agar plates from the frozen glycerol stocks and the inoculums were prepared using overnight cultures in LB broth at 37°C with appropriate aeration.

**Table 4:** Bacterial strains, plasmids and primers used in this study.

	Genotype, relevant characteristics, or sequence (5′-3′)	Source or reference
Strains		
*P. aeruginosa*		
PAO1	wild-type; phototropic	[60]
MPAOMS	*mutS::ISlacZA/hah* (MPA32417); Tc^r^	[61]
MPAOML	*mutL::ISlacZA/hah* (MPA46306); Tc^r^	[61]
Plasmids		
pMC5-*mutS*	pBBR1MCS-5 carrying *P.aeruginosa mutS*; Gm^r^	[56]
pMC5-*mutL*	pBBR1MCS-5 carrying *P.aeruginosa mutL*; Gm^r^	[62]
Primers		
*mucA*-for	GAAGCCTGACACAGCGGCAAATGC	
*mucA*-rev	CCTCAGCGGTTTTCCAGGCTGGCTGC	
*lasR*-for	CACGGGCGCATGCGCCTC	
*lasR*-rev	AAGCTTCTATATAGAAGGGCA	
*mexZ*-for	CGCGACAGTAGCATATAATC	
*mexZ*-rev	TACATCGACGGCAAGCGCCT	
*mutS*-for1	GCCCGTATGACCGACCTCT	
*mutS*-rev1	CCGAGTCGCGATCGAAGT	
*mutS*-for2	CCGCGCGCCATGGGACTTCGAT	
*mutS*-rev2	TTCGGCGAGTTCGGGATA	
*mutS*-for3	CACCACCATCGGCACCTAT	
*mutS*-rev3	GTTGGCCACGAACGGTGT	
*mutS*-for4	TGGTCGAGCAGGTGCTGG	
*mutS*-rev4	ATTCTAGCAGCTTGTGCGG	
*mutL*-for1	ACAGCCTGTCCAGCGACAAC	
*mutL*-rev1	CTCGTCTCGCGCCTCGTGCA	
*mutL*-for2	TGCACGAGGCGCGAGACGAG	
*mutL*-rev2	TAACGGCGCGAAGTAGGCCTT	
*mutL*-for3	AAGGCCTACTTCGCGCCGTTA	
*mutL*-rev3	AGGCAGGGAAGACATCGGAAC	

Tc^r^, tetracycline resistance; Gm^r^, gentamicin resistance.

### Ethics Statement

The *P. aeruginosa* isolates were obtained from the Bacteriological Laboratories of the Hospital de Niños Santísima Trinidad (Córdoba) and Hospital Alemán (Buenos Aires), Argentina, as byproducts of the routine established for bacterial typing and antimicrobial susceptibility testing. In this sense, sputa sampling was not performed with the aim to fulfill the study described in this work, and isolates recovered from these sputa were just a derivative of the habitual CF patient therapeutic controls. Thus, we obtained a collection of 38 *P.aeruginosa* isolates which was characterized in this work. It is important to clarify that bacterial isolates were processed anonymously, there was no contact with the patients or access to medical records, and that the therapeutic treatments of the patients were not modified in any case as a consequence of the results obtained in this study. The research protocols followed in this study were approved and reviewed by the Institutional Review Board of the Biological Chemistry Research Center of Córdoba (Centro de Investigaciones en Química Biológica de Córdoba, CIQUIBIC), which waived the need for a written consent from the patients involved.

### Genotyping by Pulse Field Gel Electrophoresis (PFGE)

All isolates were genotyped by PFGE using the *SpeI* enzyme as described elsewhere [54]. The clonal relatedness was determined according to Tenover *et al.* [55], who established that isolates with PFGE patterns which differed: by 1–3 bands are closely related clones; by 4–6 bands are possibly related clones; and by ≥7 bands are considered to belong to different strains.

### Determination of mutation frequency of *P. aeruginosa* CF isolates

Mutation rates determined via fluctuation analysis can provide good estimations of mutagenesis [53]. However, since our interest was merely to identify hypermutators in the collection of *P. aeruginosa* CF isolates, we performed the quantification of rifampin-resistant mutant frequencies which is considered to be proportional to the estimation of mutation frequencies. Mutation frequencies were determined as previously described [56]. Briefly, three bacterial colonies of each *P. aeruginosa* isolate were cultured in LB medium at 37°C for 24 h with shaking performed at 225 r.p.m. Appropriate dilutions of the cultures were plated on LB agar plates to determine the total number of viable cells, or on LB agar supplemented with 100 µg ml^−1^ rifampin to count the number of rifampin-resistant cells, following incubation overnight at 37°C. Then, the mutation frequency was determined as the ratio of the number of rifampin–resistant cells and the number of viable cells. Strains were considered hypermutators when their mutation frequencies were ≥2×10^−7^, which corresponds to a mutation frequency approximately 20 fold higher than that obtained from the reference *P. aeruginosa* PAO1 strain (2.4±1.3×10^−8^) and which has been shown to score for strong MRS-deficient mutators [19]. Determinations were carried out in duplicate for two independent experiments, and the results were expressed as means with their standard deviations.

### Sequence analysis of *mucA*, *lasR*, *mexZ*, *mutS* and *mutL* genes

Primers for PCR amplification and DNA sequencing of *mucA*, *lasR*, *mexZ*, *mutS* and *mutL* genes are shown in Table 4. PCR amplifications were performed under the following conditions: 8 min at 95°C, 33 cycles of 1 min at 94°C, 1 min 20 sec at 60°C (for *mucA*, *mexZ, mutS* and *mutL*) or at 50°C (*lasR*) 2 min at 72°C, and a final extension of 10 min at 72°C. The PCR products were cleaned with a Gel Purification kit (QIAGEN) and both strands were directly sequenced by using their respective PCR primers (Macrogene Corp., USA). To score for mutations within the genes, the sequencing results were compared with the corresponding gene sequences of the PAO1 strain (www.pseudomonas.com) by using the BLAST program of the NCBI database (www.ncbi.nlm.nih.gov/blast/).

The analyses of the linkage between hypermutability and mutations observed in *mucA*, *lasR* and *mexZ* included only nonsynonymous mutations, which are expected to cause partial or complete loss of function in the genes by generation of premature stop codons, shifts of reading frames or deletions/insertions in the coding sequence. To predict the effect of nonsynonymous (missense) mutations on protein function, the SIFT software was [57] used with default parameters (median conservation of sequences = 3; removal of sequences >90% identical to the query). Mutations were considered as “not tolerated” when *P*<0.05.

### Complementation assays with the *mutS* and *mutL* genes

To investigate the genetic basis for the hypermutator phenotype, the hypermutator isolates were complemented with the cloned *P. aeruginosa* wild-type *mutS* and *mutL* genes (Table 4). Vector pBBR1MC-5 [58], which harbors *mutS* or *mutL* genes, was successively transferred into the different hypermutator *P. aeruginosa* CF isolates by electroporation as described by Choi and Schweizer [59]. The transformants were selected on LB agar plates supplemented with 100 µg of gentamicin ml^−1^, and the complementation was checked by the rifampin test as described above. For each isolate, complementation was verified for three independent transformant colonies.

### Antibiotic susceptibility tests and resistant mutant subpopulation quantification

The inhibition-zone diameters of the thirty eight *P. aeruginosa* CF isolates were determined for imipenem, meropenem, ceftazidime, ciprofloxacin and tobramycin in Mueller-Hilton (MH) agar plates by using conventional disks (Rosco). Approximately 0.5 McFarland standard suspensions were used for the inocula standardization of each isolate, and the diameters of the inhibition zones were measured after 24 h and 48 h of incubation at 37°C. The relative number (<10, 10 to 100, >100) of resistant mutant subpopulations growing within the inhibition zones was determined for each antibiotic after 48 h of incubation at 37°C, as described previously [43]. *P. aeruginosa* PAO1 strain and the hypermutable MPAOMS and MPAOML strains (Table 4) were used as controls. All assays were performed in duplicate.

Antibiotic multiresistance was defined as resistance to at least three antibiotics.

### Statistical analysis

Statistical analyses were performed using Fisher's exact test, considering a *P* value of >0.05 as significant.
